# Women’s knowledge and attitudes related to cervical cancer and cervical cancer screening in Isiolo and Tharaka Nithi counties, Kenya: a cross-sectional study

**DOI:** 10.1186/s12885-018-4642-9

**Published:** 2018-07-18

**Authors:** Murithi Gatumo, Susan Gacheri, Abdul-Rauf Sayed, Andrew Scheibe

**Affiliations:** 1 Community Health Access Program (CHAP), P O Box 2756, Nairobi, 00202 Kenya; 2 Bristol-Myers Squibb Foundation (BMSF), Woodmead office park, Johannesburg, South Africa

**Keywords:** Cross-sectional study, Kenya, Cervical cancer, Knowledge and attitudes, HPV

## Abstract

**Background:**

Cervical cancer is the most common cancer among women in Kenya. However, only 3% of women are routinely screened. This study aimed to assess women’s knowledge and attitudes towards cervical cancer and cervical cancer screening in Kenya’s Isiolo and Tharaka Nithi counties.

**Methods:**

A cross-sectional survey was conducted between January and March 2017. Using a multistage cluster sampling methodology, 451 women 18 years of age and older participated in the study. Interviewers administered a 35-item questionnaire collecting demographic information, knowledge of risk factors and attitudes towards cervical cancer and cervical cancer screening. Bivariate and multivariate analyses of cervical cancer knowledge and demographic characteristics were conducted.

**Results:**

The response rate for the study was 98% (451/460). Two-thirds of the study participants originated from Tharaka Nithi county (*n* = 318). Respondents reported a median age of 32; 70.5% were married; and 35.0% had primary education. Eighty percent of the participants were aware of cervical cancer, 25.6% of whom had previously undergone a cervical screening examination, and 44.4% had above-average knowledge of risk factors of cervical cancer. Knowledge of cervical cancer risk factors was significantly associated with employment status (adjusted odds ratio = 1.6; 95% CI: 1.0–2.6) and county of origin (adjusted odds ratio = 2.8; 95% CI: 1.6–5.0). Almost all (89.2%) of those who had heard of cervical cancer categorised it as “scary”. There was a marginal significant difference in the overall attitude assessment score towards cervical cancer between participants from Isiolo and Tharaka Nithi counties; the mean (SD) score was 2.13 (0.34) and 2.20 (0.30) respectively. The score was comparatively higher among participants residing in Tharaka Nithi (95% CI: 0.002–0.146; *p* = 0.043).

**Conclusions:**

Interventions to increase cervical cancer knowledge are needed in Isiolo and Tharaka Nithi counties, Kenya. Additional research is needed to further understand and assess the effectiveness of different strategies to improve attitudes regarding cervical cancer in order to increase the uptake of screening services, particularly among less-educated women and those in hard-to-reach areas.

**Electronic supplementary material:**

The online version of this article (10.1186/s12885-018-4642-9) contains supplementary material, which is available to authorized users.

## Background

Globally, cervical cancer is the fourth most frequent cancer among women [1]. In 2012, there were approximately 530,000 new cases of cervical cancer and 270,000 related deaths; the majority occurring in low- and middle-income countries. Worldwide, the highest incidence rates of cervical cancer occur in eastern, western and southern Africa, with age-standardised rates of 34.5, 33.7 and 26.8 cases per 100,000 population, respectively [1]. This high burden of disease is largely a result of lack of access to screening services and inadequate screening uptake due to female patients’ limited knowledge or fears about cervical cancer screening [2–4]. Research has also suggested that a lack of male involvement may be an overlooked obstacle to cervical cancer screening [5]. In resource-poor settings, it is estimated that less than 5 % of women are screened for cervical cancer compared to 40.0 to 50.0% in high-income countries [6].

The 2014 World Cancer Report notes that vaccination against human papilloma virus (HPV) (the virus responsible for most cervical cancers) along with early detection and treatment services are key interventions to decrease cervical cancer incidence [6–8].

In Kenya, cancer is estimated to be the third leading cause of death after infectious and cardio-vascular diseases. Cancer accounts for 7.0% of overall national mortality [6]. The annual incidence of cancer is close to 37,000 new cases with an annual mortality of over 28,000. The leading cancers in women are cervix (40.1/100,000), breast (38.3/100,000) and oesophageal cancer (15.1/100,000) [7].

Cervical cancer poses a great burden on women’s health in Kenya due to its high incidence and the poor prognosis of most patients. Data from qualitative and health-facility based research has provided insights into reasons for cervical cancer screening practices in Kenya. Low screening coverage has been attributed to several factors, including limited access to and availability of screening services, screening cost, lack of trained service providers, inadequate equipment and supplies, inadequate monitoring and evaluation of screening programmes, and a health service system that is overwhelmed by health demands [9, 10]. Although community awareness of cervical cancer may have grown because of the introduction of the cervical cancer screening programmes and HPV vaccine in select areas of Kenya, low levels of knowledge and awareness, fears relating to speculum examination, discomfort with male health workers, and limited spousal approval, have been identified as additional factors contributing to suboptimal screening rates [9–11].

Little is known about women’s knowledge and attitudes around cervical cancer and cervical cancer screening in the eastern part of Kenya. This semi-arid region has high poverty levels, low education levels among women of reproductive age and limited sexual and reproductive and cancer health services.

The objective of this study was to determine the knowledge and attitudes of cervical cancer and cervical cancer screening and prevention among women aged 18 years and above in the Isiolo and Tharaka Nithi counties of eastern Kenya.

## Methods

A baseline cross-sectional quantitative survey of women’s knowledge and attitudes towards cervical cancer and cervical cancer screening was conducted between January and March 2017 in Isiolo and Tharaka Nithi counties in Kenya. The survey was carried out in these counties before the start of a cervical cancer awareness and screening project.

### Study setting

Isiolo and Tharaka Nithi counties have estimated populations of 143,294 and 365,330, respectively. The inhabitants of Isiolo county belong to several ethnic groups, the majority of whom are pastoralists. The population of Tharaka Nithi county are largely the Ameru and engage in mixed agricultural farming [12].

Isiolo county is considered a ‘hard-to-reach’ area. Accessibility is hampered by poor infrastructure, insecurity occasioned by conflicts among inhabitants (due to limited water supply, pasture and other reasons) and with neighbouring countries (Ethiopia and Somalia). This has contributed to economic instability, demonstrated by the uncharacteristically high poverty levels (63.0% for Isiolo county and 49.0% for Tharaka Nithi county versus the national level of 46.0%) [13].

Despite the government’s provision of free primary education and subsidised secondary education, literacy levels are low (59.8% in Isiolo county and 71.2% in Tharaka Nithi county versus the national average of 66.0% in 2013) [14]. The 2014 Kenyan Demographic Health Survey highlighted that in Isiolo county, 39.7% of women of reproductive age lacked formal education compared to 11.4% of their male counterparts. In Tharaka Nithi county, approximately one-third of women and men of reproductive age had some primary education [12]. The median age at first marriage in Isiolo county (18.5 years) is lower than the other counties of Kenya’s Eastern region (20.5 years) [12].

Isiolo county has one referral hospital and 27 health facilities with a physician-to-population ratio of 1:143,000. In 2014, two-thirds of women opted for a home delivery [8]. In contrast, Tharaka Nithi county has three district hospitals, one sub-district hospital and 84 health facilities. In 2014, the physician-to-population ratio was 1:21,000 [11] and 77.7% of women delivered in a health facility [12].

### Population

Females aged 18 years old and older at the time of enrolment, residing in the specified counties for at least six months prior to data collection, were considered eligible for participation in the study. Women with cervical cancer diagnosed before taking part in the study were considered ineligible for participation.

### Sample size

The sample size was calculated using the formula for estimating a population proportion *n* = *p*(1 − *p*)(1.96)^2^ ÷ *d*^2^ [15]*.* The expected proportion (p) of Kenyan women with adequate or comprehensive knowledge of cervical cancer was 50% (*p* = 0.5), with a desired precision of 7.0% (d = 0.07). Since this study utilised a multistage cluster sampling method, the sample size was multiplied by the design effect of 2. The sample size calculation also took into consideration a non-response rate of 10.0%. Therefore, the minimum sample size required was 431. Ultimately, a sample of 460 was used to gain sufficient statistical power to explore possible demographic factors associated with knowledge of cervical cancer.

### Sampling method

Participants were selected using the multistage cluster sampling technique. This sampling method is effective in geographically dispersed populations. The method eliminates the need for a complete list of all units (households) in the population, and ensures that selected population units will be closer together, thus costs for personal interviews are reduced, and fieldwork simplified [16]. A proportional stratified sample was drawn from the respective counties. Random samples of 30 sub-locations were selected from each county. The households from each sub-location were selected by the systematic random sampling method [17]. The interviewers adhered to a predetermined sampling interval. Only one woman aged 18 years or older per household was interviewed. When an eligible respondent was not available during the first visit, an interviewer arranged alternative visits to complete data collection procedures.

### Questionnaire

There is no validated questionnaire to assess knowledge and attitudes related to cervical cancer specifically in Kenya. For the purpose of this study, questions to assess attitudes towards cervical cancer were adapted from other validated breast cancer questionnaires including Champion’s Health Belief Model Scale and Powe Fatalism Inventory (modified version) [18–21]. Questions were chosen based on their relevance to the Kenyan cultural setting, considering the diversity of cultural and religious beliefs in Kenya. The questionnaire consisted of 8 closed-ended questions that assess knowledge of risk factors and 16 closed-ended questions that assess attitudes related to cervical cancer. The questionnaire was initially developed in English and then translated into the local language (Swahili).

The paper-based questionnaire contained sections to capture demographic characteristics, knowledge and attitudes towards cervical cancer and cervical cancer screening. Trained interviewers administered the questionnaire. In cases where the interviewer spoke the same local language as the respondent, questions were asked in the local language. One pilot session of the questionnaire was done in each of five ethnic communities to ensure women respondents were able to understand it and that questions were being interpreted as intended.

All of the questions used to assess knowledge of cervical cancer risk factors in the questionnaire were considered to be true. Knowledge scores for these questions were coded as ‘1’ for a correct response (“Yes”) and ‘0’ for an incorrect (“No”) or ‘not sure’ response. A composite score was derived for each of the 8 questions. A respondent who achieved a composite score greater than or equal to 4 (≥50%) was considered as knowledgeable (average and above), otherwise not [22, 23]. Attitude was assessed on a scale of 1 to 3 (yes / not sure / no, respectively). A negative response was assigned a score of ‘1’; not sure ‘2’; and a positive response ‘3’. An average score was calculated for each respondent from the sum total of 16 questions. The questionnaire is provided in Additional file 1.

The Kuder–Richardson Formula 20 (KR-20) [24] reliability coefficients and Cronbach’s alpha [25] coefficients were calculated for dichotomously scored variables and variables scored on a scale of 1 to 3, respectively. The KR-20 coefficient for the group of questions pertaining to knowledge of risk factors for cervical cancer was 0.71. Values greater than or equal to 0.70 were considered acceptable [26]. Similarly, the Cronbach’s alpha showed acceptable reliability for the group of questions pertaining to attitude assessment of cervical cancer, which was 0.75.

### Data analysis

Data was captured in EpiData 3.1 [27] and exported to Stata 13.1 [28] for statistical analysis. Categorical variables are presented as frequency tables, and numerical variables as descriptive measures, expressed as median and range. The association between knowledge of cervical cancer (yes/no) and demographic characteristics was assessed using bivariate and multivariate logistic regression analysis. Odds ratios (OR) were used to test the association between binary variables and 95% confidence intervals (CI) that did not span unity were considered as thresholds of statistical significance. Adjusted odds ratios (aOR) were used in multivariate analysis.

### Ethical considerations

Ethical clearance for this study was provided by the Isiolo and Tharaka Nithi County Health Departments (ethics committee reference: ICDH/NGO.1VOL.1/35) in January 2017. Participants provided written consent to participate in the study. Confidentiality was ensured throughout the process of data collection and analysis through the use of de-identified code numbers. Participants were not remunerated for participation.

## Results

### Demographic characteristics

A total of 451 women participated in the study, 29.5% from Isiolo county and 70.5% from Tharaka Nithi county, giving a total response rate of 98%. The median age of participants was 32 (ranging between 18 to 85 years) and approximately two-thirds (66.3%) were aged 18 to 39 years (Table 1). The majority were married (70.5%) and half (50.8%) were employed or self-employed. Thirty-five percent of the respondents had a primary level of education while 14.2% were non-literate. There were significant differences in the demographic characteristics between the study participants residing in the two counties (Table 1). The participants in Tharaka Nithi county were significantly older, 64.5% were over the age of 29 years of age compared to the participants in Isiolo county (51.1%) (OR = 1.7; 95% CI: 1.1–2.7). Over half of the participants in Tharaka Nithi (54.1%) had attained primary level of education compared to the participants in Isiolo (25.6%) (OR = 3.4; 95% CI: 2.1–5.5). A significant proportion of Tharaka Nithi women were employed or self-employed (62.6%) compared to the women in Isiolo (22.6%) (OR = 5.7; 95% CI: 3.5–9.5).

**Table 1:** County of origin and demographic characteristics of study participants, by county (*n* = 451)

	County of origin		
	Isiolo	Tharaka Nithi	Total
	*N* = 133	*N* = 318	*N* = 451
Variable	N (%)	N (%)	N (%)
Age (years)			
< 20	10 (7.5)	14 (4.4)	24 (5.3)
20–29	55 (41.4)	99 (31.1)	154 (34.2)
30–39	38 (28.6)	83 (26.1)	121 (26.8)
40–49	13 (9.8)	58 (18.2)	71 (15.7)
50–59	8 (6.0)	37 (11.6)	45 (10)
≥60	9 (6.8)	27 (8.5)	36 (8)
Education level			
Non-literate	47 (35.3)	17 (5.4)	64 (14.2)
Read and write	14 (10.5)	9 (2.8)	23 (5.1)
Primary	38 (28.6)	120 (37.7)	158 (35)
High school	30 (22.6)	121 (38.1)	151 (33.5)
Diploma and above	4 (3.0)	51 (16.0)	55 (12.2)
Marital status			
Married	99 (74.4)	219 (68.9)	318 (70.5)
Single	18 (13.5)	77 (24.2)	95 (21.1)
Divorced	7 (5.3)	12 (3.8)	19 (4.2)
Widowed	9 (6.8)	10 (3.1)	19 (4.2)
Employment status			
Housewife	72 (54.1)	43 (13.5)	115 (25.5)
Employed/self-employed	30 (22.6)	199 (62.6)	229 (50.8)
Unemployed	29 (21.8)	59 (18.6)	88 (19.5)
Student	2 (1.5)	17 (5.4)	19 (4.2)

*n* number of respondents

*%* percentage

### Knowledge assessment of cervical cancer

Overall, 79.8% (360/451) of the study participants were aware of cervical cancer, and 15.1% (68/451) had heard of HPV. Among those who were aware of cervical cancer, 83.6% (301/360) had heard of cervical cancer screening and 25.6% (92/360) had undergone a cervical cancer screening examination. Those who were aware of cervical cancer reported that their primary sources of information were from family or friends (45.0%, *n* = 162), a health care facility (40.3%, *n* = 145), radio/television (40.6%, *n* = 146), and less than 6.0% (*n* = 20) stated social media, newspaper or a non-governmental organisation. Fewer than two-thirds of those who had heard about cervical cancer gave the appropriate response to two of the eight questions on risk factors for cervical cancer; cervical cancer is preventable (61.9%, 223/360) and having many different sexual partners is a risk factor (61.1%, 220/360). One in six participants (16.9%, 61/360) knew that HPV is a risk factor for cervical cancer (Table 2).

**Table 2:** Knowledge of cervical cancer risk factors among participants who were aware of cervical cancer (*n* = 360)

	Frequency of correct responses	
Questions to assess knowledge of risk factors for cervical cancer^a^	n	%
Is cervical cancer preventable?	223	61.9
Is having many different sexual partners a risk factor for cervical cancer?	220	61.1
Is oral contraception a risk factor for cervical cancer?	211	58.6
Is smoking a risk factor for cervical cancer?	198	55.0
Is HIV a risk factor for cervical cancer?	141	39.2
Are you more likely to get cervical cancer if your family has it?	88	24.4
Is giving birth to many babies a risk factor for cervical cancer?	63	17.5
Is human papilloma virus (HPV) a risk factor for cervical cancer?	61	16.9

*n* number of respondents

*%* percentage

^a^ The correct response for these questions was “Yes”

As described in the methodology, using the composite score for knowledge, the results showed that fewer than half (44.4%) of the participants who were aware of cervical cancer had above-average knowledge of risk factors for cervical cancer. A significant association between the outcome variable (knowledgeable of risk factors for cervical cancer (yes/no)) and selected demographic variables (education, employment status and county of origin) was observed in the bivariate analysis. Only employment status and county of origin were significant predictors of knowledge when adjusted for all of the demographic variables in Table 3. Women who were employed were almost twice as likely to be knowledgeable of cervical cancer (aOR = 1.6; 95% CI: 1.0–2.6) compared to unemployed women, and women in Tharaka Nithi were almost three times more likely to be knowledgeable of cervical cancer (aOR = 2.8; 95% CI: 1.6–5.0) compared to women from Isiolo (Table 3).

**Table 3:** Associations between demographic characteristics and knowledge of risk factors for cervical cancer among participants who were aware of cervical cancer (*n* = 360)

Demographic variables	Knowledgeable of risk factors for cervical cancer		OR	*p*-value	aOR
	No (%)	Yes (%)	(95% CI)		(95% CI)
Age (years)					
18–29	85 (42.5%)	59 (36.9%)			
≥30	115 (57.5%)	101 (63.1%)	1.3 (0.8–2.0)	0.279	1.2 (0.7–1.9)
Education level					
≤ Primary education	108 (54.0%)	66 (41.2%)			
≥ Secondary education	92 (46.0%)	94 (58.8%)	1.7 (1.1–2.6)	0.016	1.3 (0.8–2.1)
Marital status					
Unmarried	58 (29.9%)	53 (33.1%)			
Married	142 (71.0%)	107 (66.9%)	0.8 (0.5–1.3)	0.339	0.8 (0.5–1.3)
Employment status					
Unemployed/student	110 (55.0%)	55 (34.4%)			
Employed	90 (45.0%)	105 (65.3%)	2.3 (1.5–3.7)	< 0.001	1.6 (1.0–2.6)^a^
County of origin					
Isiolo	75 (37.5%)	22 (13.8%)			
Tharaka Nithi	125 (62.5%)	138 (86.2%)	3.8 (2.2–6.7)	< 0.001	2.8 (1.6–5.0)^a^

*OR* odds ratio

*aOR* adjusted odds ratio

*n* number of respondents

*%* percentage

^a^Statistically significant

### Attitude assessment of cervical cancer and cervical screening

Attitudes towards cervical cancer were assessed separately using 16 questions (Table 4). Almost all (89.2%) of those who had heard of cervical cancer categorised it as “scary”. Over half of the women responded that “cervical cancer would threaten a relationship with her husband, boyfriend or partner” (56.7%) and also preferred a female health worker to conduct a cervical examination (55.8%). Nearly two-thirds (61.4%) of respondents perceived the examinations to be positive and believed that “health care workers performing cervical examinations are not rude to women”. There was a marginal significant difference in the overall attitude assessment score towards cervical cancer between participants from Isiolo and Tharaka Nithi counties; the mean (SD) score was 2.13 (0.34) and 2.20 (0.30) respectively. The score was comparatively higher among participants residing in Tharaka Nithi (95% CI: 0.002–0.146; *p* = 0.043).

**Table 4:** Attitude assessment of cervical cancer among participants who had heard of cervical cancer (*n* = 360)

Questions to assess attitudes related to cervical cancer	Yes		Not sure		No	
	n	%	n	%	n	%
My chances of getting cervical cancer in the next few years are high.	85	23.6	66	18.3	209	58.1
I feel I will get cervical cancer some time during my life.	54	15	61	16.9	245	68.1
The thought of cervical cancer scares me.	321	89.2	8	2.2	31	8.6
Problems I would experience with cervical cancer would last a long time.	274	76.1	23	6.4	63	17.5
Cervical cancer would threaten a relationship with my boyfriend, husband or partner.	204	56.7	37	10.3	119	33.1
If I developed cervical cancer, I would not live longer than 5 years.	172	47.8	41	11.4	147	40.8
Having cervical exams takes too much time.	43	11.9	119	33.1	198	55
Having cervical exams is too painful.	71	19.7	142	39.4	147	40.8
Health care workers doing cervical exams are rude to women.	34	9.4	105	29.2	221	61.4
I have other problems more important than having cervical exams in my life.	66	18.3	12	3.3	282	78.3
I am too old to have cervical exams regularly.	29	8.1	8	2.2	323	89.7
There is no health centre close to my house to have cervical exams.	100	27.8	21	5.8	239	66.4
If there is cancer development in my destiny, having cervical exams will not prevent it.	140	38.9	27	7.5	193	53.6
I prefer a female health worker to conduct cervical exams.	201	55.8	20	5.6	139	38.6
I will never have cervical exams if I have to pay for it.	145	40.3	11	3.1	204	56.7
I would be ashamed to lie on a gynaecologic examination table and show my private parts during a cervical exam.	61	16.9	4	1.1	295	81.9

*n* number of respondents

*%* percentage

## Discussion

To our knowledge this is the first published study to assess the knowledge and attitudes about cervical cancer among women in Isiolo and Tharaka Nithi counties, Kenya. Findings highlight low levels of knowledge and negative attitudes towards cervical cancer in these counties. The study highlighted that the majority (80.0%) of female study participants had never undergone a cervical screening exam. These factors are likely contributing to cervical cancer-related morbidity and mortality in this part of Eastern Kenya.

The study sample was comparable by age, marital status and education level to women sampled in the 2014 Kenyan Demographic Health Survey [12]. Older women, and women with higher levels of education had better knowledge of the risk factors of cervical cancer, which is consistent with previous studies in sub-Saharan Africa [22, 29–32].

Patients living in poor, rural communities, especially in low-income countries, often seek medical attention when cancer is advanced [33]. In Kenya, low levels of knowledge have been associated with late presentation of cervical cancer [34]. Late diagnosis leads to poor prognosis and needs to be urgently addressed [35]. Improving cervical cancer awareness and addressing negative attitudes around cervical cancer screening are crucial components of an effective cervical cancer prevention programme. Data from a Kenyan cohort study supports the potential role of increased awareness of cervical cancer in HPV vaccine uptake [36]. This is important as the country considers introducing the HPV vaccine to the national vaccination programme [37].

Women in Tharaka Nithi county were almost three times more likely to have heard about cervical cancer compared with women from Isiolo county. Higher literacy levels, lower poverty levels, peace, higher number of health facilities and health professional density could be contributing to the better levels of knowledge of cancer among women in that county [13, 14]. Furthermore, civil society organisations have been implementing community-based family planning and cervical cancer awareness activities in Tharaka Nithi county since 2010, while no similar programmes exist in Isiolo county [38].

The high levels of negative attitudes and fear towards cervical cancer in both counties is unsurprising and perhaps an appropriate response, given the high rates of late-stage diagnosis of the disease and increased mortality seen in similar populations. A previous study among women at a Kenyan teaching hospital noted that fear of abnormal results and lack of finances were common barriers to cervical cancer screening (22.4 and 11.4%) [39].

Family and friends are the most important source of information, followed by healthcare facilities and radio/ television. Evidence of effective interventions to enhance the uptake of cervical cancer screening services in Africa is limited [40]. A randomised controlled trial on targeted health talks at government health clinics in rural Kenya did not improve cervical cancer screening uptake [41]. However, smaller pre-post assessments of an educational movie [42], peer delivered health talks at church services [43] and market places [41] in Nigeria have increased knowledge, attitudes and perceptions among women about cervical cancer and cervical cancer screening, and could be effective in the Kenyan context. The integration of health awareness themes into popular television and radio dramas have been carried out in cervical cancer awareness campaigns in other African countries with mixed results [18, 42] but remains a potentially important method of health promotion in rural low-educated communities.

### Limitations

Our sample was predominantly rural and may not be generalisable to other populations. The inclusion of more women who were educated, working and married may limit the generalisability of findings to women in these areas who are less educated, unemployed and/ or single. We did not include questions around symptom awareness, which could have provided insights into potential associations with knowledge, attitudes and timely presentation. The self-report nature, although facilitated, might have caused bias and over- or under-estimation of certain variables such as lack of equivalent local terminology for medical words such as HPV. Understanding of the questions among people with first languages other than Swahili may have affected the responses provided, particularly in Isiolo county, where several other languages are spoken. We did not collect data on the languages in which each interview was conducted, but the interviewers were trained on study procedures and were fluent in Swahili and relevant local languages. The influence of religion on knowledge and attitudes was not assessed and could be another influencing factor. Isiolo county is predominantly Muslim while Tharaka Nithi county is predominantly Christian [17]. This variance might have influenced access to information and attitudes around cervical cancer however, previous research has not documented this association. Although the Cronbach’s alpha for the attitude scale was acceptable, we used unvalidated measures for the measurement of attitudes and knowledge, which may have affected the psychometric properties of the measure and is another limitation of our study.

### Future research

Future studies that assess cervical cancer knowledge and attitudes should consider including questions around symptoms to explore ways for timely presentation at health services. Additional research to further understand and assess the effectiveness of different strategies to increase cervical cancer knowledge, improve attitudes and increase uptake of cervical cancer screening services is needed.

## Conclusion

This study found that the overall knowledge of risk factors for cervical cancer among women in Isiolo and Tharaka Nithi counties was low. Lack of awareness of cervical cancer and knowledge of risk factors are likely barriers to accessing cervical cancer screening services and related care. These barriers should be addressed through novel multi-faceted strategies that could include the use of peer-education, mass media and interventions delivered at healthcare facilities and by community health workers. However, approaches should be tailored to each county to account for the different contexts and evaluated for effectiveness.

## Additional file

Additional file 1: The file includes the questions incuded in the study around interview information; demographic information, as well as knowledge and attitudes assessments. (DOCX 40 kb)

## Abbreviations

aOR: Adjusted odds ratio
CI: Confidence interval
HPV: Human papilloma virus
KR-20: Kuder–Richardson Formula 20
OR: Odds ratio
