# Supplementary material for: Women’s knowledge and attitudes related to cervical cancer and cervical cancer screening in Isiolo and Tharaka Nithi counties, Kenya: a cross-sectional study
Source: BMC Cancer. 2018 Jul 18;18:745. doi: 10.1186/s12885-018-4642-9 (PMC6052645; doi:10.1186/s12885-018-4642-9)
Supplement: Supplementary file 1 — The file includes the questions incuded in the study around interview information; demographic information, as well as knowledge and attitudes assessments. (DOCX 40 kb) [file 12885_2018_4642_MOESM1_ESM.docx]

**CHAP COUNTY QUESTIONNAIRE**

**BASELINE SURVEY**

Knowledge and Attitudes of cervical cancer screening and prevention among women residing in Isiolo and Tharaka- Nithi Counties, Kenya

Respondents should be women 18 years and above

| **SECTION 1: Interview Information** | | | **Code** |
| --- | --- | --- | --- |
|  | Participant‘s code |  |  |
|  | Name of County |  |  |
|  | Name of Sub-County |  |  |
|  | Name of Location |  |  |
|  | Name of Sub-Location |  |  |
|  | Date of interview | DD / MM / YYYY | DD / MM / YYYY |
|  | Full name of interviewer |  |  |

| **SECTION 2: Demographic information** | | | **Code** |
| --- | --- | --- | --- |
|  | Age in years |  |  |
|  | Education level | 1. Non-literate 2. Read & Write 3. Primary education 4. High school 5. Technical 6. Diploma 7. University degree |  |
| 2.3 | Marital status | 1. Single 2. Married 3. Divorced 4. Widowed |  |
| 2.4 | Occupation | 1. Housewife 2. Employed/self employed 3. Unemployed 4. Student 5. Other: Specify |  |
| 2.5 | Religion | 1. Protestant 2. Catholic 3. Islam 4. Other: Specify |  |
|  | How many children? |  |  |

| **SECTION 3: Knowledge assessment of Cervical Cancer** | | | | | | | **Code** |
| --- | --- | --- | --- | --- | --- | --- | --- |
|  | Have you ever heard of cervical cancer?  **If No or not sure, go to section 4** | *Yes* | *No* | | *Not sure* | |  |
|  | What is your source of information about cervical cancer?  *Multiple responses are possible*  (Tick the appropriate response) | Health care facility | | | | |  |
|  |  | Word of mouth (friends / family) | | | | |  |
|  |  | Radio | | | | |  |
|  |  | Television | | | | |  |
|  |  | Social media | | | | |  |
|  |  | Newspaper | | | | |  |
|  |  | NGO | | | | |  |
|  |  | Other (specify) | | | | |  |
|  | Have you ever HEARD of cervical cancer screening? | *Yes* | | *No* | | *Not sure* |  |
|  | Have you ever HAD a cervical cancer screening or examination? | *Yes* | | *No* | | *Not sure* |  |
|  | Have you heard of human papillomavirus or HPV vaccine? | *Yes* | | *No* | | *Not sure* |  |
| **Questions related to risk factors (a cause of) for Cervical Cancer** | | | | | | | |
|  | Are you more likely to get cervical cancer if someone in your family has it? | *Yes* | | *No* | | *Not sure* |  |
|  | Is human papilloma virus (HPV) a risk factor for cervical cancer? | *Yes* | | *No* | | *Not sure* |  |
|  | Is HIV a risk factor for cervical cancer? | *Yes* | | *No* | | *Not sure* |  |
|  | Is smoking a risk factor for cervical cancer? | *Yes* | | *No* | | *Not sure* |  |
|  | Is oral contraception a risk factor for cervical cancer? | *Yes* | | *No* | | *Not sure* |  |
|  | Is cervical cancer preventable? | *Yes* | | *No* | | *Not sure* |  |
|  | Is having many different sexual partners a risk factor for cervical cancer? | *Yes* | | *No* | | *Not sure* |  |
|  | Is giving birth to many babies a risk factor for cervical cancer? | *Yes* | | *No* | | *Not sure* |  |

| **SECTION 4: Attitude assessment of Cervical Cancer** | | | | | |
| --- | --- | --- | --- | --- | --- |
| 4.1 | My chances of getting cervical cancer in the next few years are high | *Yes* | *No* | *Not sure* |  |
| 4.2 | I feel I will get cervical cancer some time during my life | *Yes* | *No* | *Not sure* |  |
| 4.3 | The thought of cervical cancer scares me | *Yes* | *No* | *Not sure* |  |
| 4.4 | Problems I would experience with cervical cancer would last a long time. | *Yes* | *No* | *Not sure* |  |
| 4.5 | Cervical cancer would threaten a relationship with my boyfriend, husband or partner. | *Yes* | *No* | *Not sure* |  |
| 4.6 | If I developed cervical cancer, I would not live longer than 5 years. | *Yes* | *No* | *Not sure* |  |
| 4.7 | Having Cervical exams takes too much time | *Yes* | *No* | *Not sure* |  |
| 4.8 | Having Cervical exams is too painful | *Yes* | *No* | *Not sure* |  |
| 4.9 | Health care workers doing Cervical exams are rude to women | *Yes* | *No* | *Not sure* |  |
| 4.10 | I have other problems more important than having Cervical exams in my life | *Yes* | *No* | *Not sure* |  |
| 4.11 | I am too old to have Cervical exams regularly | *Yes* | *No* | *Not sure* |  |
| 4.12 | There is no health centre close to my house to have Cervical exams | *Yes* | *No* | *Not sure* |  |
| 4.13 | If there is cancer development in my destiny, having Cervical exams will not prevent it | *Yes* | *No* | *Not sure* |  |
| 4.14 | I prefer a female health worker to conduct Cervical exams | *Yes* | *No* | *Not sure* |  |
| 4.15 | I will never have Cervical exams if I have to pay for it | *Yes* | *No* | *Not sure* |  |
| 4.16 | I would be ashamed to lie on a gynaecologic examination table and show my private parts to have a Cervical exam | *Yes* | *No* | *Not sure* |  |
